# Policy brief: how can rehabilitation be strengthened in Switzerland? Evidence-informed policy considerations based on the 2023 World Health Assembly resolution on strengthening rehabilitation in health systems

**DOI:** 10.3389/phrs.2026.1609467

**Published:** 2026-09-15

**Authors:** Roxanne Maritz, David Weisstanner, Gerold Stucki, Oliver Stoller, Anke Scheel-Sailer, Christian Sturzenegger, Carla Sabariego, The Center for Rehabilitation in Global Health Systems, The Advisory Board of the LIFE Forum Rehabilitation

**Affiliations:** 1 Center for Rehabilitation in Global Health Systems, Faculty of Health Sciences and Medicine, University of Lucerne, Lucerne, Switzerland; 2 Competence Centre for Rehabilitation Engineering and Science, ETH Zurich, Zürich, Switzerland; 3 Center for Rehabilitation and Sports Medicine, Inselspital and Berner Reha Zentrum, Bern University Hospital, University of Bern, Bern, Switzerland; 4 Bellikon Rehabilitation Clinic, Bellikon, Switzerland; 5 Swiss Paraplegic Research, Nottwil, Switzerland; 6 LIFE Forum Rehabilitation, Lucerne Initiative for Functioning, Health and Well-being, Faculty of Health Sciences and Medicine, University of Lucerne, Lucerne, Switzerland

**Keywords:** rehabilitation, health policy, health systems, Switzerland, World Health Assembly Resolution

## Abstract

**Background:**

Rehabilitation needs in Switzerland are rising due to population ageing and the growing prevalence of non-communicable diseases. Yet, these needs are not comprehensively met and rehabilitation remains underrepresented in national strategies. Given the 2023 WHA Resolution on Rehabilitation, there is a growing need to understand how rehabilitation can be strengthened in Switzerland.

**Analysis:**

A national stakeholder survey identified challenges for strengthening rehabilitation, including financing and workforce issues, limited integration into primary care, and interprofessional collaboration, while main opportunities focused on policy development and advocacy efforts. Based on these findings, experts developed practical policy considerations through structured workshops and a consolidation survey.

**Policy considerations:**

The 22 considerations call for high-level commitment through a National Swiss Rehabilitation Alliance, financing reforms that value rehabilitation as investment, and expansion of affordable, high-quality services across all care levels, among others. They emphasize coordinated care pathways, quality mechanisms, workforce development, modern data systems, stronger research and knowledge translation, and equitable access to assistive technologies.

**Conclusion:**

These considerations provide practical directions for integrating rehabilitation into national health priorities and advancing Swiss implementation of the WHA Resolution.

## Background

Rehabilitation has been defined by the World Health Organization (WHO) as “*a set of interventions designed to optimize functioning in individuals with health conditions or impairments in interaction with their environment*” [1]. It allows persons with health conditions to keep or regain their independence in daily and social activities, work or education and is associated to faster hospital discharges, lower care dependency and lower healthcare costs [2, 3]. In Switzerland, rehabilitation is not only recognized as a key health intervention but is also anchored in the Federal Constitution: Article 112b mandates the Confederation and the Cantons to promote rehabilitation of persons eligible for invalidity benefits [4].

Global demographic and epidemiological trends are driving a surge in rehabilitation needs. Rapid population ageing and the increasing prevalence of non-communicable diseases (NCDs) are central drivers, with the Global Burden of Disease (GBD) reporting remarkable increases in rehabilitation needs worldwide [5]. In recognition of this, all member states of the World Health Assembly (WHA), including Switzerland, unanimously passed in 2023 a Resolution on Strengthening Rehabilitation in Health Systems (hereafter called the “Resolution”) [6]. The Resolution urges member states to address nine key action areas, including raising national commitment to rehabilitation; strengthening financing mechanisms; expanding rehabilitation services to all levels of healthcare; and ensuring the integrated and coordinated provision of high-quality, affordable, accessible, gender-sensitive, appropriate, evidence-based rehabilitation interventions along the continuum of care, among others.

In Switzerland, the need for rehabilitation is also growing. In 2022, over one-third of the Swiss population was living with a chronic health condition [7]. NCDs are the leading cause for premature deaths and linked to about one-third of the health costs in Switzerland [8]. A recent study, using GBD estimates, reported that four out of nine people in Switzerland could benefit from rehabilitation and that Switzerland experienced an increase of 38.2% in Years Lived with Disability (YLD) between 1990 and 2021 (see Figure 1; [9]). Nevertheless, rehabilitation seems not yet acknowledged as a key health strategy worth being integrated in the current Swiss national health strategy (Health2030) [10] or in the Swiss NCDs-Strategy [8]. While the latter focuses on prevention, Health2030 focuses on long-term care (LTC). Therefore, the potential of rehabilitation to support the response to demographic and epidemiological trends remains insufficiently explored.

**FIGURE 1:**
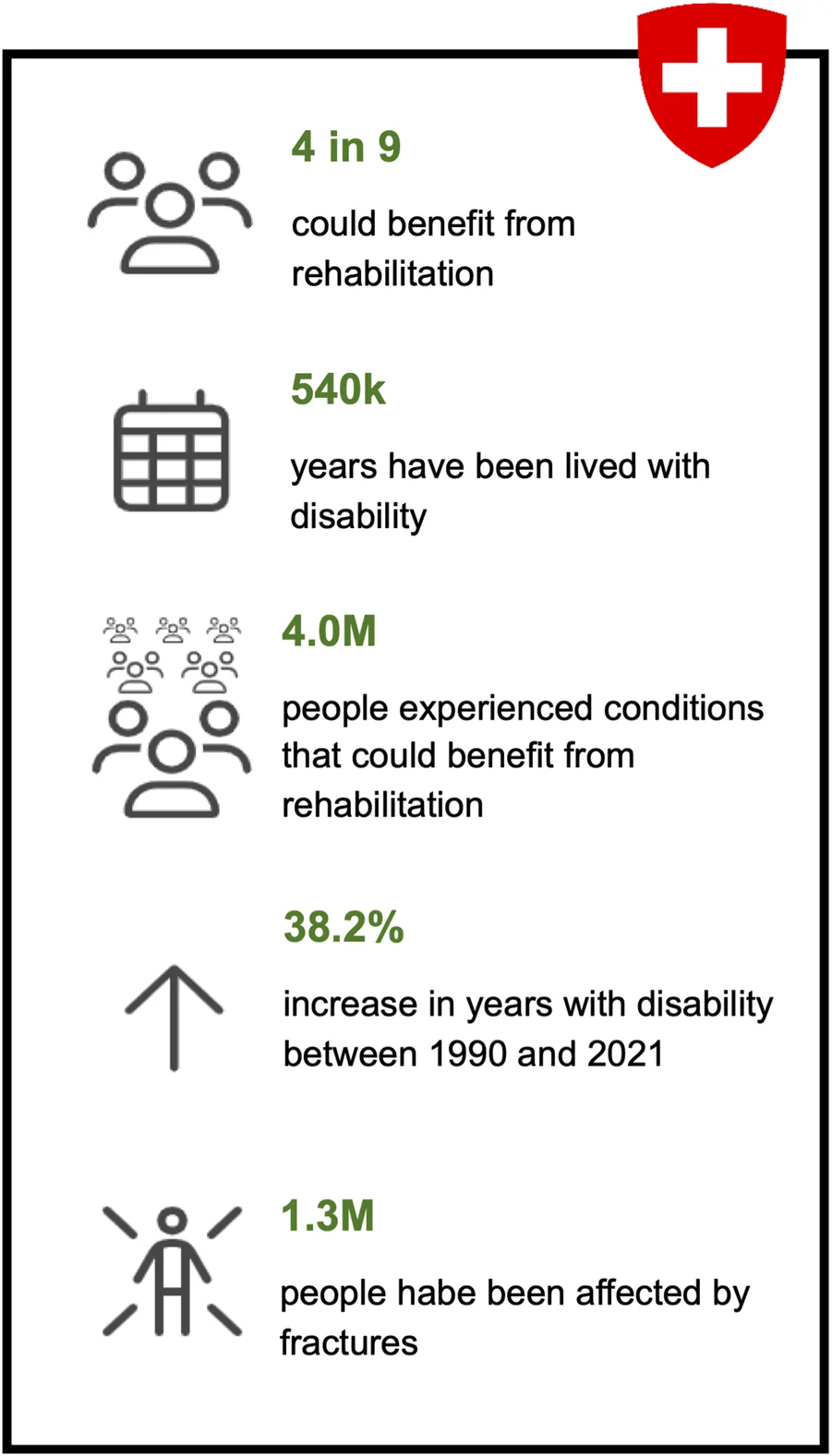
Estimated rehabilitation needs in Switzerland based on Global Burden of Disease data (Institute for Health Metrics and Evaluation, GBD 2021, available at https://viz-archive.healthdata.org/rehabilitation/, University of Washington, US, 2021, Policy brief: how can rehabilitation be strengthened in Switzerland?, Switzerland, 2025).

The objective of this policy brief is to present evidence-informed policy considerations for strengthening rehabilitation in Switzerland to inform the implementation of the actions of the WHA Resolution for rehabilitation. The policy brief builds on recent Swiss evidence, including qualitative studies and a representative national stakeholder survey [11, 12]. Based on these findings, experts - including members and advisory board members of the Center for Rehabilitation in Global Health Systems [13] and the Lucerne Initiative for Functioning, Health and Wellbeing (LIFE) [14] - co-designed a set of policy considerations.

## Analysis

Evidence about the challenges impeding a better integration of rehabilitation in the Swiss health system has grown in recent years. Spiess et al. [12] assessed the perspectives of healthcare professionals and unveiled a misalignment between the current reimbursement system and the growing cases complexity, the lack of community rehabilitation services and the poor integration of rehabilitation in primary care were pointed out as challenges. To overcome these and further problems the development and implementation of an action plan on national level was suggested [12].

Capitalizing on the momentum brought by the Resolution, a representative survey was conducted in Switzerland with a broader range of rehabilitation stakeholders including healthcare professionals, patients, health and accident insurance representatives, staff from educational and health information institutions [11]. The survey addressed the relevance of the nine key action areas of the Resolution for Switzerland, as well as challenges and opportunities for strengthening rehabilitation in the Swiss health system. All key action areas were considered highly relevant, especially the development of multidisciplinary rehabilitation skills, integrated and coordinated interventions and strengthening of the financial mechanisms.

Together, these studies highlight three consistent challenges. First, low political recognition as rehabilitation is not yet sufficiently politically prioritised in national strategies [8, 10]. Second, system fragmentation as responsibilities and financing vary across cantons and insurance schemes, creating inequities in access [11, 12]. Third, capacity limitations since workforce shortages, lack of community-based services, and weak information systems hinder service delivery [11, 12].

### Development of policy considerations

Preliminary policy considerations (PCs) were derived from findings of the national stakeholder survey and informed by previous Swiss rehabilitation research [11]. These preliminary PCs were then discussed, amended, and prioritized by experts in two half-day in-person workshops. Participants included members of two existing expert panels: Members and advisory board Center for Rehabilitation in Global Health Systems (CRGHS, *n* = 31) and the LIFE Forum Rehabilitation advisory board (*n* = 14). All invited experts were given the opportunity to contribute during the workshop and consultation phases. Following these workshops, the revised PCs were circulated to all experts via an online consultation survey for validation and further input. Feedback was analyzed thematically using an inductive coding approach to refine the final set of PCs. Where differing perspectives emerged, revisions were discussed within the project team and incorporated when consensus could be reached. The final wording of the policy considerations was informed by both workshop discussions and feedback received through the consultation survey. Ethical approval was obtained from the Ethics Board of the University of Lucerne (IRB #2025-018), and the study protocol was published previously [15]. An overview of the methodological steps and timeline is provided in Figure 2.

**FIGURE 2:**
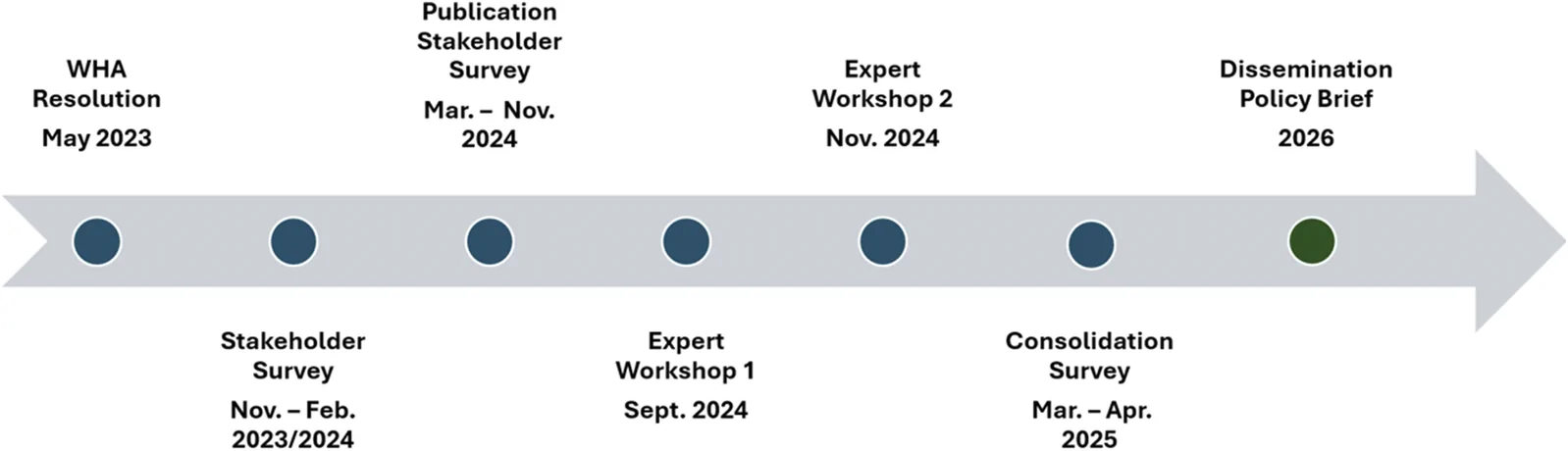
Development of the policy considerations for strengthening rehabilitation in Switzerland (Policy brief: how can rehabilitation be strengthened in Switzerland?, Switzerland, 2025).

The resulting policy considerations should be interpreted as evidence-informed recommendations derived from stakeholder perspectives and expert consensus and not as empirical findings.

## Policy considerations

The following five thematic areas synthesize the 22 detailed PCs (Tables 1–5). Supplementary Appendix 1 provides the complete list of PCs, including the original wording of the WHA Resolution, related best practice examples identified by the experts, and translations of all considerations into German and French. The policy considerations are intended to provide strategic directions for strengthening rehabilitation in Switzerland and should not be interpreted as a detailed implementation roadmap.

**TABLE 1:** Policy considerations 1-2: Strengthening governance and national commitment (Policy brief: how can rehabilitation be strengthened in Switzerland?, Switzerland, 2025).

WHA action area	Policy considerations
1: Raise awareness and build national commitment	1) Establish a **National Swiss Rehabilitation Alliance** to unite key stakeholders around the shared goal of advocating for the strengthening of rehabilitation as a core health service within Switzerland’s national health priorities. These stakeholders may include policymakers, healthcare professionals, rehabilitation professional associations, health and social insurers, healthcare users, advocacy groups, and academic partners. The Alliance will promote a shared understanding of the definition, scope, and benefits of rehabilitation, emphasising its role not only in recovery but also in enabling participation, improving functioning, and supporting healthy ageing. A key objective of the Alliance should be to launch an inclusive, multilingual national rehabilitation campaign aimed at the general public. 2) Establish a **Coordination Unit for Rehabilitation** within the Swiss national health authorities. The overarching aim of this Coordination Unit is to strengthen rehabilitation in the Swiss health system. It should serve as a central source of information, coordinate efforts, and support the development and implementation of rehabilitation strategies. It should be located within the Federal Office of Public Health (BAG/OFSP) and/or the Swiss Conference of the Cantonal Ministers of Public Health (GDK/CDS). To ensure the integration of rehabilitation policy and financing across health and social systems, authorities such as the Federal Social Insurance Office (BSV/OFAS), the Federal Statistical Office (FSO), the State Secretariat for Economic Affairs (SECO), Cantonal Invalidity Insurance Offices (IV/AI), and the Conference of Invalidity Insurance Offices (IVSK/COAI), should also be considered for involvement. Furthermore, the Coordination Unit for Rehabilitation serves as a contact point to the WHO.

**TABLE 2:** Policy considerations 3-9: Reform financing and expand access to services (Policy brief: how can rehabilitation be strengthened in Switzerland?, Switzerland, 2025).

WHA action area	Policy considerations
2: Strengthen financing mechanism	3) Review **tariff systems and health insurance policies** to ensure fair, accessible and sustainable rehabilitation services that are based on evolving population needs. This includes addressing potential financial barriers for healthcare users such as out-of-pocket costs, including deductibles and co-payments. Efforts should support comprehensive coverage in inpatient setting, extend it to outpatient settings, and strengthen the connection between health and social service financing. Additionally, supportive financing mechanisms throughout the entire patient journey can then be ensured. 4) Promote **rehabilitation as a high-value, cost-effective investment** in the health system. This can be achieved by adopting innovative service models such as tele-rehabilitation, home-based rehabilitation, and coordinated care approaches that emphasise goal setting and case management to improve efficiency. Further return-on-investment (ROI) studies should be conducted to assess the value for money delivered by different rehabilitation models. These evaluations should consider both clinical and societal outcomes, such as improved participation, reduced burden, and return to work, in order to capture the full value generated by rehabilitation services. 5) Align **investments in rehabilitation** with regional and cantonal projections of healthcare needs, taking demographic trends into account. This alignment should be informed by standardised indicators and needs assessments.
3: Expand to all levels of the health system	6) Ensure **availability of rehabilitation services across all levels of care** by strengthening primary care, including general practitioners, nurses, and allied healthcare professionals and by extending services to the community level (e.g. tele-rehabilitation and mobile teams) and the social service system. Highlight the role of rehabilitation in secondary and tertiary prevention and chronic disease management to improve long-term outcomes and reduce the burden on the health system. This can be supported by integrating rehabilitation into ongoing cantonal initiatives and improving regional alignment. 7) Prioritise **affordable, high-quality and accessible rehabilitation services in all regions**, focusing on addressing existing geographic and social disparities. Pay particular attention to vulnerable groups, such as older adults (aged 65+), people with disabilities or chronic diseases, unemployed individuals or caregivers, those living in rural or remote areas or individuals with a migration background. Ensure equitable access to specialised rehabilitation care regardless of residence, income, insurance status, culture, language, or other potential barriers also by aligning related cantonal and regional initiatives.
4: Ensure integrated and coordinated interventions	8) Create **measures and regulations to promote the alignment of inpatient, outpatient, and community-based rehabilitation services across the continuum of care**. These should include quality standards and interoperable digital systems to facilitate communication and coordination between different levels of care. Furthermore, transitions of care management (e.g. hospital to home, inpatient to outpatient) and vocational integration services should be considered to avoid care gaps. 9) Invest in **strong support mechanisms to ensure high-quality rehabilitation care**, building on the work of the Federal Quality Commission (FQC) and related initiatives, such as the Swiss National Association for Quality Development (ANQ). These mechanisms may include training programmes, audit systems, and patient feedback tools. Quality indicators should be rehabilitation-specific and reflect patient-centred outcomes, including the operationalisation and measurement of participation goals.

**TABLE 3:** Policy considerations 10-12: Build and retain a skilled rehabilitation workforce (Policy brief: how can rehabilitation be strengthened in Switzerland?, Switzerland, 2025).

WHA action area	Policy considerations
5: Develop multidisciplinary rehabilitation skills	10) Embed **rehabilitation into the core curriculum of all healthcare professions, from basic to specialised and continuing education**. This should include foundational knowledge for all healthcare professionals, as well as advanced competencies for those working directly in rehabilitation. Emphasise multidisciplinary and interprofessional rehabilitation education, particularly for general practitioners, who often serve as key entry points into the rehabilitation system. Enhance the effectiveness of multidisciplinary rehabilitation teams by strengthening case management and coordination skills to ensure continuity of care and effective collaboration across care settings. These educational components should be structurally embedded through collaboration with academic institutions and professional associations. 11) Address **workforce shortages by ensuring sufficient numbers of trained professionals in all rehabilitation disciplines**, paying particular attention to the needs of an ageing population and the rise in chronic conditions. Support the development of national workforce monitoring and forecasting tools for evidence-based planning, and consider implementing incentive programmes (e.g. scholarships, job placements, and professional development opportunities) to attract and retain rehabilitation professionals, particularly in underserved areas. Additionally, provide support for the accreditation and training of foreign-trained providers to strengthen the rehabilitation workforce. 12) Improve **retention and working conditions in order to maintain a stable, healthy, and skilled rehabilitation workforce**. This includes developing attractive, flexible new working models, including options for professionals returning to the field. Monitoring staff satisfaction and turnover rates can inform ongoing improvements and support sustainable workforce development.

**TABLE 4:** Policy considerations 13-19: Strengthen data, research, and knowledge translation (Policy brief: how can rehabilitation be strengthened in Switzerland?, Switzerland, 2025).

WHA action area	Policy considerations
6: Enhance information systems and data collection	13) Invest in **health information systems to improve the systematic collection, sharing, and use of rehabilitation-related data, including functioning information**. Functioning, as defined by the International Classification of Functioning, Disability and Health (ICF), describes an individual’s lived experience of health. It refers to the intrinsic health capacities of an individual and their actual performance in real-life contexts, as operationalised through the domains of body functions and structures, activities, and participation, in interaction with contextual factors. Explore the potential of artificial intelligence to support data collection, analysis, decision-making, and service improvement. This includes ensuring the critical, responsible and ethically grounded use of AI with a strong emphasis on data protection and privacy. 14) Promote the **recognition of functioning as a third core health indicator, alongside morbidity and mortality,** by providing evidence and building consensus among stakeholders. As a valuable indicator of population health and an important component for a robust monitoring of rehabilitation outcomes, functioning should be integrated into national health information systems and national statistics. 15) Address **digital infrastructure gaps by ensuring data security and promoting the adoption of the national electronic health record (EHR) system** that reports on a core set of rehabilitation indicators and outcomes in a unified and interoperable way. Affordable solutions, such as open-source platforms or subsidies for small providers, should be supported to facilitate broad adoption.
7: Promote rehabilitation research	16) Expand **high-quality rehabilitation research at system, service and clinical levels** to evaluate the effectiveness and cost-effectiveness of interventions. This includes implementation research to bridge the gap between evidence and practice, as well as the development and adoption of standardised, patient-centred instruments to improve case coordination along the continuum of care and to allow for the systematic documentation of rehabilitation outcomes, including individual goals. 17) Establish **rehabilitation science as an academic focus** with designated full professorships for rehabilitation science and for different professional disciplines, such as rehabilitation medicine, physical therapy, occupational therapy, etc. 18) Strengthen the **evidence base for rehabilitation and focus on the translation of research findings into health services and policy**, through national guidelines, expert advisory groups, and structured knowledge translation strategies. Additionally, support health systems and policy research to inform decision-making, including through dedicated research funding mechanisms. 19) Promote **national collaboration and establish rehabilitation research networks**, including cantons, universities, insurers, and other stakeholders, to increase their reach and impact. Unite existing rehabilitation science and management initiatives from universities and universities of applied sciences within their multidisciplinary programmes.

**TABLE 5:** Policy considerations 20-22: Ensure equity and preparedness through assistive technology and emergency integration (Policy brief: how can rehabilitation be strengthened in Switzerland?, Switzerland, 2025).

WHA action area	Policy considerations
8: Integrate in emergency preparedness and response	20) Fully **integrate rehabilitation into emergency response plans and preparedness strategies**, with a focus on digital tools for real-time coordination during and after crises. To maintain continuity of care during and after public health emergencies, rehabilitation should not be left out in disaster committees. Rehabilitation professionals should be included in training, simulations, and emergency exercises. Furthermore, rehabilitation should be integrated into long-term coping processes following crises, public health emergencies, or other emergencies.
9: Invest in assistive technology	21) Expand **efforts to ensure equitable access to cost-effective assistive devices and technologies, as well as environmental modifications**, to improve functioning in daily life and autonomy. Access should be based on individual needs rather than diagnosis, with a focus on populations at risk of requiring long-term care and individuals with disabilities across all age groups. Assess and strengthen access across insurance schemes and patient groups, removing barriers related to eligibility and cost. This should include a broad range of assistive technologies such as mobility aids, communication tools, and digital solutions, to ensure inclusive and comprehensive support. User training and support must be provided to ensure effective and sustained use. 22) **Educate healthcare providers and users about the availability, use, and benefits of assistive devices and technologies**. Guidance and support should be offered across all care settings, including hospitals, primary care, and long-term care facilities. Ensure that information about assistive devices and technologies is easily accessible to patients and caregivers to support informed use, increase acceptance, and promote self-management and independence.

### Strengthen governance and national commitment

Rehabilitation remains insufficiently prioritized in Swiss health strategies. Establishing a National Swiss Rehabilitation Alliance (PC1) and a Coordination Unit for Rehabilitation (PC2) within federal authorities could improve visibility, leadership, and alignment with international efforts. Stronger governance would enable coordinated planning and investment across cantons (Table 1).

### Reform financing and expand access to services

Current reimbursement systems inadequately support complex rehabilitation needs. Reviewing tariffs and insurance policies to reduce out-of-pocket costs and integrate health and social financing (PC3) is essential. Rehabilitation should be recognized as a cost-effective investment (PC4) and planned according to demographic and cantonal needs (PC5). Expanding service availability across care levels (PC6, PC7), improving care pathways (PC8), and strengthening quality mechanisms (PC9) would enhance equity and efficiency (Table 2).

### Build and retain a skilled rehabilitation workforce

Workforce shortages and uneven distribution require embedding rehabilitation in all health curricula (PC10), improving workforce monitoring and incentives (PC11), and enhancing working conditions and career opportunities (PC12). Expanding interprofessional training would strengthen coordinated care and professional recognition (Table 3).

### Strengthen data, research, and knowledge translation

Limited functioning data constrain evidence-based planning. Integrating rehabilitation indicators into interoperable health information systems (PC13–PC15) and promoting functioning as a core health metric would improve monitoring. Expanding research capacity and strengthening knowledge translation mechanisms (PC16–PC18) and establishing related networks (PC19) are essential to link evidence with policy and practice (Table 4).

### Ensure equity and preparedness through assistive technology and emergency integration

Rehabilitation should be included in emergency preparedness and disaster response (PC20–PC21) to maintain service continuity. Expanding equitable access to assistive technologies and strengthening provider and user education (PC22) would advance inclusion and preparedness across all populations (Table 5).

## Conclusion

Recent evidence confirms that rehabilitation is highly relevant for Switzerland [11] and increasingly needed due to the rising prevalence of NCDs, population ageing, and longer years lived with disability [7–9]. Yet rehabilitation remains largely absent from both the Health2030 Strategy [10] and the Swiss NCD Strategy [8], limiting coordinated investment and access, and potentially increasing long-term costs and care dependencies.

Switzerland nonetheless has strong foundations for progress. Ongoing initiatives in financing reform and tele-rehabilitation [16–18], national quality frameworks such as the Swiss National Association for Quality Development (ANQ) [19] and Swiss Reha [20], and expanding interprofessional education [21–23] demonstrate readiness for system-level integration.

The 2023 WHA Resolution [6] provides a clear global mandate and our synthesis of national evidence and expert perspectives [11, 12] shows that all nine Resolution action areas apply to Switzerland. The 22 policy considerations, grouped into five thematic areas, namely governance, financing and access, workforce, data and research, and equity and preparedness, outline practical steps for strengthening rehabilitation in Switzerland.

Implementing these considerations will require coordinated action across federal and cantonal authorities, insurers, healthcare providers, professional associations, academic institutions and healthcare users. Given the federal structure of the Swiss health system, prioritisation and phased implementation may be necessary. Existing regional variations in service availability, financing arrangements, and access to rehabilitation across cantons further highlight the importance of addressing equity considerations during implementation. Acting now would position Switzerland as a leader in implementing the WHA Resolution on strengthening rehabilitation in health systems and in advancing rehabilitation within high-income health systems.

## Group members of the Center for Rehabilitation in Global Health Systems

Christian Baumgartner (University of Lucerne), Melanie Behr (REHAB Basel), Jerome Bickenbach (Swiss Paraplegic Research), Stefan Boes (University of Lucerne), Mirjam Brach (Swiss Paraplegic Research), Thekla Brunkert (University of Lucerne), Mira Burri (University of Lucerne), Stephanie Christensen (Health Department of the Canton of Basel-Stadt), Nicola Diviani (Swiss Paraplegic Research), Claudia Gabriel (Lucerne Cantonal Hospital), Stefan Goetz (Clinic St. Katharinental, Spital Thurgau AG), Dorothee Guggisberg (Lucerne University of Applied Sciences and Arts), Sarah Mantwill (University of Lucerne), Luise Menzi (Bern University of Applied Sciences), Thomas Nyffeler (University of Lucerne), Diana Pacheco Barzallo (University of Lucerne), Claudio Perret (Swiss Paraplegic Research), Birgit Prodinger (University of Augsburg), Robert Riener (ETH Zurich), Sara Rubinelli (University of Lucerne), Bernhard Rütsche (University of Lucerne), Urban Schwegler (Swiss Paraplegic Research), Vanessa Seijas (University of Lucerne).

## Group members of the Advisory Board LIFE Forum Rehabilitation

Melanie Behr (REHAB Basel), Stephanie Christensen (Health Department of the Canton of Basel-Stadt), Claudia Gabriel (Neurorehabilitation, Lucerne Cantonal Hospital), Stefan Goetz (Clinic St. Katharinental, Spital Thurgau AG), Josef Grab (Suva), Dorothee Guggisberg (Lucerne University of Applied Sciences and Arts), Thomas Nyffeler (University of Lucerne), Klaus Pleyer (formerly Clinic Davos), Claudia Roesle (Clinic Lengg AG), Gianni Rossi (Suva Clinics; Clinique Romande de Réadaptation / Rehaklinik Bellikon), Urban Schwegler (Swiss Paraplegic Research), Diana Sigrist-Nix (Swiss Paraplegic Center), Stefan Staubli (Swiss Paraplegic Center), Michaela Tschuor (Health and Social Department, Canton of Lucerne), Adrian Wyss (XUND Bildung Gesundheit Zentralschweiz).
