# Supplementary material for: Policy brief: how can rehabilitation be strengthened in Switzerland? Evidence-informed policy considerations based on the 2023 World Health Assembly resolution on strengthening rehabilitation in health systems
Source: Public Health Rev. 2026 Sep 15;47:1609467. doi: 10.3389/phrs.2026.1609467 (PMC13619533; doi:10.3389/phrs.2026.1609467)
Supplement: Supplementary file 1 [file Table1.docx]

**Appendix 1 Policy Considerations and Best Practice Examples to Strengthen Rehabilitation in Switzerland**

**ENGLISH VERSION**

| **WHA Resolution Action Area** | **Original Resolution Call** | **Policy Considerations** | **Best Practice Examples** |
| --- | --- | --- | --- |
| 1. Raise awareness and build national commitment | Member states are urged to raise awareness of and build national commitment for rehabilitation, including for assistive technology, and strengthen planning for rehabilitation, including its integration into national health plans and policies, as appropriate, while promoting interministerial and intersectoral work and meaningful participation of rehabilitation users, particularly persons with disabilities, older persons, persons in need of long-term care, community members, and community-based and civil society organizations at all stages of planning and delivery. | 1) Establish a **National Swiss Rehabilitation Alliance** to unite key stakeholders around the shared goal of advocating for the strengthening of rehabilitation as a core health service within Switzerland’s national health priorities. These stakeholders may include policy makers, healthcare professionals, rehabilitation professional associations, health and social insurers, healthcare users, advocacy groups, and academic partners. The Alliance will promote a shared understanding of the definition, scope, and benefits of rehabilitation, emphasising its role not only in recovery but also in enabling participation, improving functioning, and supporting healthy ageing. A key objective of the Alliance should be to launch an inclusive, multilingual national rehabilitation campaign aimed at the general public.  2) Establish a **Coordination Unit for Rehabilitation** within the Swiss national health authorities. The overarching aim of this Coordination Unit is to strengthen rehabilitation in the Swiss health system. It should serve as a central source of information, coordinate efforts, and support the development and implementation of rehabilitation strategies. It should be located within the Federal Office of Public Health (BAG/OFSP) and/or the Swiss Conference of the Cantonal Ministers of Public Health (GDK/CDS). To ensure the integration of rehabilitation policy and financing across health and social systems, authorities such as the Federal Social Insurance Office (BSV/OFAS), the Federal Statistical Office (FSO), the State Secretariat for Economic Affairs (SECO), Cantonal Invalidity Insurance Offices (IV/AI), and the Conference of Invalidity Insurance Offices (IVSK/COAI), should also be considered for involvement. Furthermore, the Coordination Unit for Rehabilitation serves as a contact point to the WHO. | - On a global level, there is a World Rehabilitation Alliance (WRA) as a network of stakeholders committed to supporting the implementation of the Rehabilitation 2030 Call for Action. (1)  - Also on a global level, there is the Learning, Acting and building for Rehabilitation in Health Systems (ReLAB-HS) which offers guidance notes and research especially for low and middle-income countries (LMICs). (2)  - In Switzerland, neither an alliance nor a coordination unit for rehabilitation exist.  - During the COVID-19 pandemic, the Swiss National COVID-19 Science Task Force demonstrated the value of coordinated scientific advice for policy, underscoring the benefit of similar mechanisms for strengthening the Swiss health system. (3) |
| **2. Strengthen financing mechanism** | Member states are urged to incorporate appropriate ways to strengthen financing mechanisms for rehabilitation services and the provision of technical assistance, including by incorporating rehabilitation into packages of essential care where necessary. | 3) Review **tariff systems and health insurance policies** to ensure fair, accessible and sustainable rehabilitation services that are based on evolving population needs. This includes addressing potential financial barriers for health care users such as out-of-pocket costs, including deductibles and co-payments. Efforts should support comprehensive coverage in inpatient setting, extend it to outpatient settings, and strengthen the connection between health and social service financing. Additionally, supportive financing mechanisms throughout the entire patient journey can then be ensured.  4) Promote **rehabilitation as a high-value, cost-effective investment** in the health system. This can be achieved by adopting innovative service models such as tele-rehabilitation, home-based rehabilitation, and coordinated care approaches that emphasise goal setting and case management to improve efficiency. Further return-on-investment (ROI) studies should be conducted to assess the value for money delivered by different rehabilitation models. These evaluations should consider both clinical and societal outcomes, such as improved participation, reduced burden, and return to work, in order to capture the full value generated by rehabilitation services.  5) Align **investments in rehabilitation** with regional and cantonal projections of healthcare needs, taking demographic trends into account. This alignment should be informed by standardised indicators and needs assessments. | - The new uniform financing system of outpatient and inpatient services (EFAS) offers a good opportunity to find and address gaps in health care financing in Switzerland (4).  - Studies on health outcomes of innovative models like hybrid cardiac telerehabilitation as conducted by the Inselspital Bern (5) serve as foundation to assess cost effectiveness and return-on-investment.  - To allow for a sustainable financing strategy, detailed needs assessments have to be developed as shown by the canton of Lucerne and the joint health region Basel. (6, 7) |
| **3. Expand to all levels of the health system** | Member states are urged to expand rehabilitation to all levels of health, from primary to tertiary, and to ensure the availability and affordability of quality and timely rehabilitation services, accessible and usable for persons with disabilities, and to develop community-based rehabilitation strategies, which will allow rehabilitation to reach underserved rural, remote and hard-to reach areas, while implementing person-centred strategies and participatory, specialized and differentiated intensive rehabilitation services to meet the requirements of persons with complex rehabilitation needs. | 6) Ensure **availability of rehabilitation services across all levels of care** by strengthening primary care, including general practitioners, nurses, and allied healthcare professionals and by extending services to the community level (e.g. tele-rehabilitation and mobile teams) and the social service system. Highlight the role of rehabilitation in secondary and tertiary prevention and chronic disease management to improve long-term outcomes and reduce the burden on the health system. This can be supported by integrating rehabilitation into ongoing cantonal initiatives and improving regional alignment.  7) Prioritise **affordable, high-quality and accessible rehabilitation services in all regions**, focusing on addressing existing geographic and social disparities. Pay particular attention to vulnerable groups, such as older adults (aged 65+), people with disabilities or chronic diseases, unemployed individuals or caregivers, those living in rural or remote areas or individuals with a migration background. Ensure equitable access to specialised rehabilitation care regardless of residence, income, insurance status, culture, language, or other potential barriers also by aligning related cantonal and regional initiatives. | - The federal office of public health states primary care as a core pillar of a health system and developed an agenda for primary care to allow access to high quality care for all people in all regions of Switzerland. This agenda also includes areas of prevention, curation, rehabilitation and palliation. (8) |
| **4. Ensure integrated and coordinated interventions** | Member states are urged to ensure the integrated and coordinated provision of high-quality, affordable, accessible, gender-sensitive, appropriate and evidence-based interventions for rehabilitation along the continuum of care, including strengthening referral systems and the adaptation, provision and servicing of assistive technology related to rehabilitation, including after rehabilitation, and promoting inclusive, barrier-free environments. | 8) Create **measures and regulations to promote the alignment of inpatient, outpatient, and community-based rehabilitation services across the continuum of care**. These should include quality standards and interoperable digital systems to facilitate communication and coordination between different levels of care. Furthermore, transitions of care management (e.g. hospital to home, inpatient to outpatient) and vocational integration services should be considered to avoid care gaps.  9) Invest in **strong support mechanisms to ensure high-quality rehabilitation care**, building on the work of the Federal Quality Commission (FQC) and related initiatives, such as the Swiss National Association for Quality Development (ANQ). These mechanisms may include training programmes, audit systems, and patient feedback tools. Quality indicators should be rehabilitation-specific and reflect patient-centred outcomes, including the operationalisation and measurement of participation goals. | - Well established quality measures like ANQ (9) or the quality criteria for rehabilitation of Swiss Reha (10) are necessary to ensure a high standard of rehabilitation and health care services.  - Projects like ParaWork (11) or ParaWG (12) by the Swiss Paraplegic Center show the importance of considering the continuum of care and also reintegration into work and social environments. |
| **5. Develop multidisciplinary rehabilitation skills** | Member states are urged to develop strong multidisciplinary rehabilitation skills suitable to the country context, including in all relevant health workers; to strengthen capacity for analysis and prognosis of workforce shortages as well as to promote the development of initial and continuous training for professionals and staff working in rehabilitation services; and to recognize and respond to different types of rehabilitation needs, such as needs related to physical, mental, social and vocational functioning, including the integration of rehabilitation in early training of health professionals, so that rehabilitation needs can be identified at all levels of care. | 10) Embed **rehabilitation into the core curriculum of all healthcare professions, from basic to specialised and continuing education**. This should include foundational knowledge for all healthcare professionals, as well as advanced competencies for those working directly in rehabilitation. Emphasise multidisciplinary and interprofessional rehabilitation education, particularly for general practitioners, who often serve as key entry points into the rehabilitation system. Enhance the effectiveness of multidisciplinary rehabilitation teams by strengthening case management and coordination skills to ensure continuity of care and effective collaboration across care settings. These educational components should be structurally embedded through collaboration with academic institutions and professional associations.  11) Address **workforce shortages by ensuring sufficient numbers of trained professionals in all rehabilitation disciplines**, paying particular attention to the needs of an ageing population and the rise in chronic conditions. Support the development of national workforce monitoring and forecasting tools for evidence-based planning, and consider implementing incentive programmes (e.g. scholarships, job placements, and professional development opportunities) to attract and retain rehabilitation professionals, particularly in underserved areas. Additionally, provide support for the accreditation and training of foreign-trained providers to strengthen the rehabilitation workforce.  12) Improve **retention and working conditions in order to maintain a stable, healthy, and skilled rehabilitation workforce**. This includes developing attractive, flexible new working models, including options for professionals returning to the field. Monitoring staff satisfaction and turnover rates can inform ongoing improvements and support sustainable workforce development. | - With the prospected changes in health care towards more integrated care, individualized treatment of multimorbid patients and an ageing population, the canton of Bern and the Bernese University of applied sciences expects the health system to be more interdisciplinary, requiring according education. (13)  - The Interprofessional Education Collaborative serves as good example for curricula development to promote interprofessional learning in as preparation for future team-work in patient care. (14)  - In Switzerland, there are certificates of advanced studies focusing on interprofessional work in health, for example the CAS in Rehabilitation Management at the University of Lucerne. Universities of applied sciences like ZHAW integrate interprofessional education in the bachelor curricula of health professions like nursing, physiotherapy, occupational therapy, midwifery and health prevention and promotion. (15) |
| **6. Enhance information systems and data collection** | Member states are urged to enhance health information systems to collect information relevant to rehabilitation, including system-level rehabilitation data, and information on functioning, utilizing the International Classification of Functioning, Disability and Health, ensuring data disaggregation by sex, age, disability and any other context-relevant factor, and compliance with data protection legislation, for a robust monitoring of rehabilitation outcomes and coverage. | 13) Invest in **health information systems to improve the systematic collection, sharing, and use of rehabilitation-related data, including functioning information.** Functioning, as defined by the International Classification of Functioning, Disability and Health (ICF), describes an individual’s lived experience of health. It refers to the intrinsic health capacities of an individual and their actual performance in real-life contexts, as operationalised through the domains of body functions and structures, activities, and participation, in interaction with contextual factors. Explore the potential of artificial intelligence to support data collection, analysis, decision-making, and service improvement. This includes ensuring the critical, responsible and ethically grounded use of AI with a strong emphasis on data protection and privacy.  14) Promote the **recognition of functioning as a third core health indicator, alongside morbidity and mortality,** by providing evidence and building consensus among stakeholders**.** As a valuable indicator of population health and an important component for a robust monitoring of rehabilitation outcomes, functioning should be integrated into national health information systems and national statistics.  15) Address **digital infrastructure gaps by ensuring data security and promoting the adoption of the national electronic health record (EHR) system** that reports on a core set of rehabilitation indicators and outcomes in a unified and interoperable way. Affordable solutions, such as open-source platforms or subsidies for small providers, should be supported to facilitate broad adoption. | - The Lucerne Childrens Hospital works together with the Center for Child Health Analytics (CCHA) child treatment and child health by analysing routinely collected health care data. Data about adult patients is collected through tools like ANQ (9), the electronic health record eHealth Suisse (16) and governmental employment data (17) and serves as a digital network for health care (16). |
| **7. Promote rehabilitation research** | Member states are urged to promote high-quality rehabilitation research, including health policy and systems research. | 16) Expand **high-quality rehabilitation research at system, service and clinical levels** to evaluate the effectiveness and cost-effectiveness of interventions. This includes implementation research to bridge the gap between evidence and practice, as well as the development and adoption of standardised, patient-centred instruments to improve case coordination along the continuum of care and to allow for the systematic documentation of rehabilitation outcomes, including individual goals.  17) Establish **rehabilitation science as an academic focus** with designated full professorships for rehabilitation science and for different professional disciplines, such as rehabilitation medicine, physical therapy, occupational therapy, etc.  18) Strengthen the **evidence base for rehabilitation and focus on the translation of research findings into health services and policy,** through national guidelines, expert advisory groups, and structured knowledge translation strategies. Additionally, support health systems and policy research to inform decision-making, including through dedicated research funding mechanisms.  19) Promote **national collaboration and establish rehabilitation research networks**, including cantons, universities, insurers, and other stakeholders, to increase their reach and impact. Unite existing rehabilitation science and management initiatives from universities and universities of applied sciences within their multidisciplinary programmes. | - On a global level, the WHO Alliance for Health Policy and Systems Research promotes the generation and use of evidence to inform health policy and systems strengthening, providing a model for advancing rehabilitation research and knowledge translation. (18) |
| **8. Integrate in emergency preparedness and response** | Member states are urged to ensure timely integration of rehabilitation into emergency preparedness and response, including emergency medical teams. | 20) Fully **integrate rehabilitation into emergency response plans and preparedness strategies**, with a focus on digital tools for real-time coordination during and after crises. To maintain continuity of care during and after public health emergencies, rehabilitation should not be left out in disaster committees. Rehabilitation professionals should be included in training, simulations, and emergency exercises. Furthermore, rehabilitation should be integrated into long-term coping processes following crises, public health emergencies, or other emergencies. | NA |
| **9. Invest in assistive technology** | Member states are urged to urge public and private stakeholders to stimulate investment in the development of available, affordable and usable assistive technology and support for implementation research and innovation for efficient delivery and equitable access with a view to maximizing impact and cost-effectiveness. | 21) Expand **efforts to ensure equitable access to cost-effective assistive devices and technologies, as well as environmental modifications,** to improve functioning in daily life and autonomy. Access should be based on individual needs rather than diagnosis, with a focus on populations at risk of requiring long-term care and individuals with disabilities across all age groups. Assess and strengthen access across insurance schemes and patient groups, removing barriers related to eligibility and cost. This should include a broad range of assistive technologies such as mobility aids, communication tools, and digital solutions, to ensure inclusive and comprehensive support. User training and support must be provided to ensure effective and sustained use.  22) **Educate healthcare providers and users about the availability, use, and benefits of assistive devices and technologies.** Guidance and support should be offered across all care settings, including hospitals, primary care, and long-term care facilities. Ensure that information about assistive devices and technologies is easily accessible to patients and caregivers to support informed use, increase acceptance, and promote self-management and independence. | NA |

**Politische Handlungsempfehlungen und “Best Practice” Beispiele zur Stärkung der Rehabilitation in der Schweiz**

**DEUTSCHE VERSION**

| **WHA Resolution “Action Area”** | **Originaltext WHA Resolution** | **Politische Handlungsempfehlungen** | **“Best Practice” Beispiele** |
| --- | --- | --- | --- |
| **1. Bewusstsein schaffen und nationales Engagement fördern** | Die Mitgliedstaaten werden aufgefordert, das Bewusstsein für Rehabilitation – einschliesslich Hilfsmittel – zu stärken und ein nationales Engagement aufzubauen. Die Planung der Rehabilitation soll verbessert und, wo angezeigt, in nationale Gesundheitspläne und -politiken integriert werden. Gleichzeitig soll die interministerielle und intersektorale Zusammenarbeit sowie die wirksame Beteiligung von Nutzerinnen und Nutzern der Rehabilitation gefördert werden, insbesondere von Menschen mit Behinderungen, älteren Menschen, Personen mit Langzeitpflegebedarf, Mitgliedern der Gemeinschaft sowie gemeindebasierten und zivilgesellschaftlichen Organisationen in allen Phasen der Planung und Umsetzung. | 1) **Gründung einer** **nationalen Schweizer Rehabilitationsvereinigung**, um wichtige Stakeholder zu vereinen, welche sich für die Stärkung der Rehabilitation als zentrale Gesundheitsleistung innerhalb der nationalen Gesundheitsprioritäten der Schweiz einsetzen. Zu diesen Stakeholdern gehören politische Entscheidungsträger und Entscheidungsträgerinnen, Gesundheitsfachpersonen, Berufsverbände im Bereich der Rehabilitation, Kranken- und Sozialversicherungen, Patientinnen und Patienten, Interessenverbände und akademische Partnerinstitutionen. Die Vereinigung soll ein gemeinsames Verständnis der Definition, des Leistungsumfangs und des Nutzens der Rehabilitation fördern, insbesondere ihre Rolle für die Teilhabe, die Verbesserung der Funktionsfähigkeit und die Unterstützung gesunden Alterns. Ein wichtiges Ziel der Vereinigung sollte die Lancierung einer inklusiven, mehrsprachigen nationalen Kampagne zur Stärkung der Rehabilitation für die breite Öffentlichkeit sein.  2) **Etablierung einer Koordinationsstelle für Rehabilitation innerhalb der nationalen Gesundheitsbehörden** der Schweiz. Das übergeordnete Ziel dieser Koordinationsstelle ist die Stärkung der Rehabilitation im Schweizer Gesundheitssystem. Sie dient als zentrale Informationsstelle, koordiniert die Bemühungen und unterstützt die Entwicklung und Umsetzung von Rehabilitationsstrategien. Sie sollte beim Bundesamt für Gesundheit (BAG) und/oder bei der Schweizerischen Konferenz der kantonalen Gesundheitsdirektorinnen und -direktoren (GDK) angesiedelt sein. Um die Integration der Rehabilitationspolitik und -finanzierung in den Gesundheits- und Sozialsystemen sicherzustellen, sollten sich auch Behörden wie das Bundesamt für Sozialversicherungen (BSV), das Bundesamt für Statistik (BFS), das Staatssekretariat für Wirtschaft (SECO), die kantonalen Invalidenversicherungsstellen (IV) und die Invalidenversicherungsstellen der Kantone (IVSK) beteiligen können. Darüber hinaus fungiert die Koordinationsstelle für Rehabilitation als Kontaktstelle zur WHO. | - Auf globaler Ebene gibt es die World Rehabilitation Alliance (WRA), ein Netzwerk von Akteurinnen und Akteuren, das sich für die Umsetzung des „Rehabilitation 2030 Call for Action“ einsetzt. (1)  - Ebenfalls auf globaler Ebene existiert „Learning, Acting and Building for Rehabilitation in Health Systems“ (ReLAB-HS), das insbesondere für Länder mit niedrigem und mittlerem Einkommen (LMICs) Leitfäden und Forschungsergebnisse bereitstellt. (2)  - In der Schweiz gibt es weder eine Allianz noch eine Koordinationsstelle für Rehabilitation.  - Während der COVID-19-Pandemie zeigte die Swiss National COVID-19 Science Task Force den Nutzen einer koordinierten wissenschaftlichen Beratung für politische Entscheidungen und unterstrich den Wert ähnlicher Strukturen für das Schweizer Gesundheitssystem. (3) |
| **2. Finanzierungs­mechanismen stärken** | Die Mitgliedstaaten werden aufgefordert, geeignete Wege zur Stärkung der Finanzierungs­mecha­nismen für Rehabilitationsleistungen und zur Bereitstellung technischer Unterstützung zu entwickeln, einschliesslich der Integration der Rehabilitation in Pakete der essentiellen Versorgung, wo erforderlich. | 3) **Überprüfung der Tarifsysteme der Krankenversicherung**, um faire, zugängliche und nachhaltige Rehabilitationsleistungen zu gewährleisten, die sich an den sich wandelnden Bedürfnissen der Bevölkerung orientieren. Dazu gehört auch die Beseitigung potenzieller finanzieller Hindernisse für den Bezug von Gesundheitsleistungen. Dies betrifft insbesondere direkte Gesundheitsausgaben wie Franchise und Selbstbehalt. Die Bemühungen sollten die umfassende Versorgung im stationären Bereich erhalten und ausbauen, diese auf den ambulanten Bereich ausweiten und die Verbindung zwischen der Finanzierung von Gesundheits- und Sozialleistungen stärken. Ebenfalls sollten unterstützende Finanzierungsmechanismen für die koordinierte Versorgung und den gesamten Behandlungsverlauf sichergestellt werden.  4) **Förderung der Rehabilitation als hochwertige und kosteneffiziente Investition** **in das Gesundheitssystem**. Dies kann durch die Einführung innovativer Rehabilitationsmodelle wie Telerehabilitation, domizilbasierte Rehabilitation und koordinierten Versorgungsansätzen erreicht werden, bei denen die Festlegung von Zielen und das Case Management im Vordergrund stehen, um die Effizienz zu verbessern. Es sollten weitere Studien zur Kapitalrendite (Return on Investment) durchgeführt werden, um das Preis-Leistungs-Verhältnis verschiedener Rehabilitationsmodelle zu bewerten. Diese Bewertungen sollten sowohl klinische als auch gesellschaftliche Ergebnisse berücksichtigen, wie z. B. verbesserte Teilhabe, geringere Belastung und Rückkehr an den Arbeitsplatz, um den ganzheitlichen Wert der Rehabilitationsleistungen zu erfassen.  5) **Orientierung der Investitionen in die Rehabilitation an regionalen und kantonalen Prognosen zu den Gesundheitsbedürfnissen der Bevölkerung** unter Berücksichtigung der demografischen Entwicklung. Diese Angleichung sollte auf standardisierten Indikatoren und Bedarfsanalysen basieren. | - Das neue einheitliche Finanzierungssystem für ambulante und stationäre Leistungen (EFAS) bietet eine gute Gelegenheit, Lücken in der Gesundheitsfinanzierung in der Schweiz zu identifizieren und zu schliessen (4).  - Studien zu Gesundheitsergebnissen innovativer Modelle wie der hybriden kardialen Telerehabilitation am Inselspital Bern (5) dienen als Grundlage für die Bewertung von Kosteneffizienz und Return-on-Investment.  - Für eine nachhaltige Finanzierungsstrategie müssen detaillierte Bedarfsanalysen entwickelt werden, wie dies der Kanton Luzern und die gemeinsame Gesundheitsregion Basel zeigen. (6,7) |
| **3. Ausweitung auf alle Ebenen des Gesundheits_­_­­­systems** | Die Mitgliedstaaten werden aufgefordert, Rehabilitation auf alle Ebenen der Gesundheitsversorgung – von der Primär- bis zur Tertiärversorgung – auszuweiten und die Verfügbarkeit und Erschwinglichkeit qualitativ hochwertiger und zeitgerechter Rehabilitationsleistungen sicherzustellen. Diese sollen für Menschen mit Behinderungen zugänglich und nutzbar sein. Zudem sollen gemeindebasierte Rehabilitationsstrategien entwickelt werden, um Rehabilitation auch in unterversorgte ländliche, abgelegene und schwer erreichbare Gebiete zu bringen. | 6) **Sicherstellung von der Verfügbarkeit von Rehabilitationsleistungen auf allen Versorgungsebenen** durch Stärkung der Primärversorgung. Dies soll die Verfügbarkeit von Allgemeinärztinnen und Allgemeinärzten, Pflegepersonal und Angehörigen verwandter Gesundheitsberufe, sowie die Ausweitung der Leistungen auf die Gemeindeebene (z. B. Telerehabilitation und mobile Teams) und das Sozialsystem einschliessen.  Ebenfalls soll die Rolle der Rehabilitation in der Sekundär- und Tertiärprävention sowie im Management chronischer Erkrankungen hervorgehoben werden, um die Langzeitergebnisse zu verbessern und die Belastung des Gesundheitssystems zu verringern. Dies kann durch die Integration der Rehabilitation in laufende kantonale Initiativen und durch eine bessere regionale Abstimmung unterstützt werden.  7) **Priorisierung von erschwinglichen, hochwertigen und zugänglichen Rehabilitationsleistungen in allen Regionen** mit einem Fokus auf gesundheitliche Chancengleichheit. Besondere Aufmerksamkeit gilt dabei vulnerablen Gruppen wie älteren Menschen (ab 65 Jahren), Personen mit Behinderungen oder chronischen Erkrankungen, Arbeitslosen oder Pflegepersonen, Menschen in ländlichen oder abgelegenen Gebieten sowie Menschen mit Migrationshintergrund. Ein gleichberechtigter Zugang zu spezialisierter Rehabilitationsversorgung muss unabhängig von Wohnort, Einkommen, Versicherungsstatus, Kultur, Sprache oder anderen potenziellen Hindernissen gewährleistet werden, auch durch die Abstimmung entsprechender kantonaler und regionaler Initiativen. | - Das Bundesamt für Gesundheit (BAG) bezeichnet die Grundversorgung als tragende Säule des Gesundheitssystems und hat eine Agenda für die Grundversorgung entwickelt, um allen Menschen in allen Regionen der Schweiz den Zugang zu qualitativ hochwertiger Versorgung zu ermöglichen. Diese Agenda umfasst auch die Bereiche Prävention, kurative Versorgung, Rehabilitation und Palliative Care. (8) |
| **4. Integrierte und koordinierte Interventionen sicherstellen** | Die Mitgliedstaaten werden aufgefordert, entlang der gesamten Versorgungskette integrierte und koordinierte, qualitativ hochwertige, erschwingliche, zugängliche, geschlechtersensible, angemessene und evidenzbasierte Rehabilitationsleistungen sicherzustellen, einschliesslich der Stärkung von Überweisungssystemen sowie der Anpassung, Bereitstellung und Wartung assistiver Technologien im Zusammenhang mit Rehabilitation – auch nach Abschluss der Rehabilitation – und der Förderung inklusiver, barrierefreier Umgebungen. | 8) **Schaffung von Massnahmen und Rahmenbedingungen zur Koordination zwischen stationären, ambulanten und gemeindebasierten (community-based) Rehabilitationsleistungen über die gesamte Versorgungskette.** Diese sollten Qualitätsstandards und interoperable digitale Systeme umfassen, um die Kommunikation und Koordinierung zwischen den verschiedenen Versorgungsebenen zu erleichtern. Darüber hinaus sollten Übergänge im Versorgungsmanagement (z. B. vom Krankenhaus nach Hause, von der stationären zur ambulanten Versorgung) und berufliche Integrationsleistungen berücksichtigt werden, um Versorgungslücken zu vermeiden.  9) **Investition in starke Unterstützungsmechanismen zur Gewährleistung einer hochwertigen Rehabilitationsversorgung**, aufbauend auf der Arbeit der Eidgenössischen Qualitätskommission (EQK) und verwandten Initiativen wie dem Nationalen Verein für Qualitätsentwicklung in Spitälern und Kliniken (ANQ). Diese Mechanismen können Schulungsprogramme, Auditsysteme und Instrumente für Patientenzufriedenheit umfassen. Qualitätsindikatoren sollten rehabilitationsspezifisch sein und patientenzentrierte Ergebnisse widerspiegeln, einschliesslich der Operationalisierung und Messung von Partizipationszielen. | - Gut etablierte Qualitätsmassnahmen wie die ANQ (9) oder die Qualitätskriterien für die Rehabilitation von SW!SS REHA (10) sind notwendig, um einen hohen Standard der Rehabilitations- und Gesundheitsleistungen sicherzustellen.  - Projekte wie ParaWork (11) oder ParaWG (12) des Schweizer Paraplegiker-Zentrums zeigen die Bedeutung der Betrachtung des gesamten Versorgungskontinuums sowie der (Wieder-)Integration in Arbeits- und soziale Umfelder. |
| **5. Multidisziplinäre Kompetenzen in der Rehabilitation entwickeln** | Die Mitgliedstaaten werden aufgefordert, starke multidisziplinäre Kompetenzen in der Rehabilitation zu entwickeln, die dem jeweiligen Landeskontext entsprechen. Dies umfasst alle relevanten Gesundheitsfachpersonen, die Stärkung der Kapazitäten zur Analyse und Prognose von Personalengpässen sowie die Förderung der Entwicklung von Aus- und Weiterbildungsprogrammen für Fachkräfte in der Rehabilitation. | 10) **Integration der Rehabilitation in das Curriculum aller Gesundheitsberufe,** von der Grundausbildung bis zur Fachausbildung und Weiterbildung. Dies sollte grundlegende Kenntnisse für alle Angehörigen der Gesundheitsberufe sowie fortgeschrittene Kompetenzen für diejenigen umfassen, die direkt in der Rehabilitation tätig sind. Der Schwerpunkt soll auf eine multidisziplinäre und berufsübergreifende Ausbildung im Bereich Rehabilitation gelegt werden, insbesondere für Allgemeinärztinnen und Allgemeinärzte, die häufig als wichtiger Zugangspunkt zum Rehabilitationssystem fungieren. Verbesserung der Effektivität multidisziplinärer Rehabilitationsteams durch die Stärkung der Case Management- und Koordinationskompetenzen, um die Kontinuität der Versorgung und eine effektive Zusammenarbeit zwischen den verschiedenen Versorgungseinrichtungen sicherzustellen. Diese Bildungskomponenten sollten durch die Zusammenarbeit mit akademischen Einrichtungen und Berufsverbänden strukturell verankert werden.  11) **Behebung des Personalmangels durch Sicherstellung einer ausreichenden Zahl ausgebildeter Fachkräfte in allen Rehabilitationsbereichen**, unter besonderer Berücksichtigung der Bedürfnisse einer alternden Bevölkerung und der Zunahme chronischer Erkrankungen.  Unterstützung der Entwicklung nationaler Instrumente zur Überwachung und Prognose des Personalbedarfs für eine evidenzbasierte Planung und Erwägung der Einführung von Anreizprogrammen (z. B. Stipendien, Arbeitsvermittlung und berufliche Weiterbildungsmöglichkeiten), um Rehabilitationsfachkräfte insbesondere in unterversorgten Gebieten anzuwerben und zu halten. Darüber hinaus Unterstützung der Akkreditierung und Weiterbildung im Ausland ausgebildeter Fachkräfte, um das Personal im Rehabilitationsbereich zu stärken.  12) **Erhöhung der Berufsverweildauer und Verbesserung der Arbeitsbedingungen, um eine stabile, gesunde und qualifizierte Fachkräftebasis im Bereich der Rehabilitation zu erhalten**. Dazu gehört die Entwicklung attraktiver, flexibler neuer Arbeitsmodelle, einschliesslich Optionen für Fachkräfte, die in den Beruf zurückkehren möchten. Die Beobachtung der Mitarbeitenden-Zufriedenheit und der Fluktuationsraten kann als Grundlage für kontinuierliche Verbesserungen dienen und eine nachhaltige Personalentwicklung unterstützen. | - Angesichts der erwarteten Veränderungen im Gesundheitswesen hin zu stärker integrierter Versorgung, individualisierter Behandlung multimorbider Patientinnen und Patienten sowie einer alternden Bevölkerung gehen der Kanton Bern und die Berner Fachhochschule davon aus, dass das Gesundheitssystem interdisziplinärer werden muss, was eine entsprechende Ausbildung erfordert. (13)  - Die „Interprofessional Education Collaborative“ dient als gutes Beispiel für die Entwicklung von Curricula, um interprofessionelles Lernen als Vorbereitung auf die zukünftige Teamarbeit in der Patientenversorgung zu fördern. (14)  - In der Schweiz gibt es Weiterbildungsangebote wie Zertifikate für interprofessionelle Zusammenarbeit im Gesundheitswesen, beispielsweise das CAS in Rehabilitation Management an der Universität Luzern. Fachhochschulen wie die ZHAW integrieren interprofessionelle Ausbildung in die Bachelor-Studiengänge der Gesundheitsberufe wie Pflege, Physiotherapie, Ergotherapie, Hebammenwesen sowie Gesundheitsprävention und -förderung. (15) |
| **6. Informations-systeme und Datenerhebung verbessern** | Die Mitgliedstaaten werden aufgefordert, Gesundheitsinformations­systeme zu verbessern, um für die Rehabilitation relevante Informationen zu erfassen, einschliesslich systembezogener Daten und Informationen zur Funktionsfähigkeit unter Nutzung der Internationalen Klassifikation der Funktionsfähigkeit, Behinderung und Gesundheit (ICF). | 13) **Investitionen in Gesundheitsinformationssysteme zur Verbesserung der systematischen Erhebung, Weitergabe und Nutzung von Daten zur Rehabilitation, einschliesslich Informationen zur Funktionsfähigkeit**. Funktionsfähigkeit, wie sie in der Internationalen Klassifikation der Funktionsfähigkeit, Behinderung und Gesundheit (ICF) definiert ist, beschreibt die gelebte Gesundheitserfahrung eines Menschen. Sie bezieht sich auf die intrinsischen Gesundheitsfähigkeiten eines Menschen und seine tatsächliche Leistungsfähigkeit in realen Lebenskontexten, operationalisiert durch die Bereiche Körperfunktionen und -strukturen, Aktivitäten und Partizipation in Wechselwirkung mit Kontextfaktoren. Dafür soll das Potenzial künstlicher Intelligenz zur Unterstützung der Datenerhebung, -analyse, Entscheidungsfindung und Verbesserung der Rehabilitationsleistungen untersucht werden. Dazu gehört auch die Gewährleistung einer kritischen, verantwortungsvollen und ethisch fundierten Nutzung künstlicher Intelligenz unter besonderer Berücksichtigung des Datenschutzes und der Privatsphäre.  14) **Förderung der Anerkennung der Funktionsfähigkeit als dritter zentraler Gesundheitsindikator neben Morbidität und Mortalität,** durch Bereitstellung von wissenschaftlichen Beweisen und Konsensbildung unter den Stakeholdern. Als wertvoller Indikator für die Gesundheit der Bevölkerung, sowie als zentraler Bestandteil für eine zuverlässige Kontrolle von Rehabilitationserfolgen, sollte die Funktionsfähigkeit in nationale Gesundheitsinformationssysteme und nationale Statistiken integriert werden.  15) **Beseitigung digitaler Infrastrukturdefizite durch Gewährleistung der Datensicherheit und Förderung der Einführung des nationalen elektronischen Patientendossiers**, das einheitlich und interoperabel eine Reihe von Kernindikatoren und -ergebnissen im Bereich der Rehabilitation erfasst. Um eine breite Einführung zu erleichtern, sollten erschwingliche Lösungen wie Open-Source-Plattformen oder Subventionen für kleine Anbieter unterstützt werden. | - Das Luzerner Kinderspital arbeitet mit dem Center for Child Health Analytics (CCHA) zusammen, um die Behandlung und Gesundheit von Kindern durch die Analyse routinemässig erhobener Gesundheitsdaten zu verbessern. Daten zu erwachsenen Patientinnen und Patienten werden über Instrumente wie ANQ (9), das elektronische Patientendossier von eHealth Suisse (16) sowie staatliche Beschäftigungsdaten (17) erfasst und dienen als digitale Vernetzung im Gesundheitswesen. (16) |
| **7. Rehabilitations-forschung fördern** | Die Mitgliedstaaten werden aufgefordert, hochwertige Forschungsarbeiten zur Rehabilitation zu fördern, einschliesslich der Forschung zu Gesundheitspolitik und Gesundheitssystemen. | 16) **Ausbau der hochwertigen Rehabilitationsforschung auf System, Versorgungserbringer- und klinischer Ebene**, um die Wirksamkeit und Kosteneffizienz von Interventionen zu bewerten. Dazu gehören Implementierungsforschung zur Überbrückung der Lücke zwischen Evidenz und Praxis sowie die Entwicklung und Einführung standardisierter, patientenzentrierter Instrumente zur Verbesserung der Fallkoordination entlang der gesamten Versorgungskette und zur systematischen Dokumentation der Rehabilitationsergebnisse, einschliesslich individueller Ziele.  Ebenfalls soll die Rehabilitationswissenschaft als akademischer Schwerpunkt etabliert werden, mit eigenen Professuren für Rehabilitationswissenschaften und einem Rehabilitationsfokus für verschiedene Fachdisziplinen wie Rehabilitationsmedizin, Physiotherapie, Ergotherapie usw.  17) **Stärkung der wissenschaftlichen Grundlage für Rehabilitation mit einem Schwerpunkt auf die Umsetzung von Forschungsergebnissen in die Gesundheitspolitik**, durch nationale Leitlinien, einen Fachbeirat und strukturierte Strategien zur Wissensumsetzung. Darüber hinaus sollten Gesundheitssysteme und Politikforschung unterstützt werden, um Entscheidungsprozesse zu informieren, unter anderem durch spezielle Finanzierungsmechanismen für die Forschung.  18) **Förderung der nationalen Zusammenarbeit und Etablierung eines Netzwerks der Rehabilitationsforschung**, darunter Kantone, Universitäten, Versicherungen und andere Stakeholder, um deren Reichweite und Wirkung zu erhöhen. Bündeln bestehender Initiativen im Bereich Rehabilitationswissenschaft und -management von Universitäten und Fachhochschulen im Rahmen ihrer multidisziplinären Programme.  19) **Förderung der nationalen Zusammenarbeit und Etablierung eines Netzwerks der Rehabilitationsforschung**, darunter Kantone, Universitäten, Versicherungen und andere Stakeholder, um deren Reichweite und Wirkung zu erhöhen. Bündeln bestehender Initiativen im Bereich Rehabilitationswissenschaft und -management von Universitäten und Fachhochschulen im Rahmen ihrer multidisziplinären Programme. | - Auf globaler Ebene fördert die WHO Alliance for Health Policy and Systems Research die Nutzung von Forschungsergebnissen zur Stärkung von Gesundheitssystemen und dient als Vorbild für den Ausbau der Rehabilitationsforschung und des Wissenstransfers. (18) |
| **8. Integration in die Notfallvorsorge und -reaktion** | Die Mitgliedstaaten werden aufgefordert, die Rehabilitation zeitnah in die Notfallvorsorge und -reaktion zu integrieren, einschliesslich in die Teams für medizinische Notfälle. | 20) **Vollständige Integration der Rehabilitation in Notfallpläne und Vorsorgestrategien,** mit Schwerpunkt auf digitale Instrumente für die Echtzeitkoordination während und nach Krisen. Um die Kontinuität der Versorgung während und nach Notfällen im Bereich der öffentlichen Gesundheit zu gewährleisten, sollte die Rehabilitation im Katastrophenschutz nicht ausser Acht gelassen werden.  Rehabilitationsfachkräfte sollten in Schulungen, Simulationen und Notfallübungen einbezogen werden. Darüber hinaus sollte die Rehabilitation in langfristige Bewältigungsprozesse nach Krisen, Notfällen im Bereich der öffentlichen Gesundheit oder anderen Notfällen integriert werden. |  |
| **9. Investitionen in Hilfsmittel und**  **assistive Technologien** | Die Mitgliedstaaten werden aufgefordert, öffentliche und private Akteure zu Investitionen in die Entwicklung verfügbarer, erschwinglicher und nutzerfreundlicher assistiver Technologien zu bewegen und die Unterstützung von Implementierungsforschung und Innovation für eine effiziente Bereitstellung und einen gerechten Zugang zu fördern. | 21) **Verstärkung der Bemühungen um einen gleichberechtigten Zugang zu kostengünstigen Hilfsmitteln und Technologien sowie zu Umbaumassnahmen,** um die Alltagsfunktionen und die Selbstständigkeit zu verbessern. Der Zugang sollte sich an den individuellen Bedürfnissen und nicht an der Diagnose orientieren, wobei der Schwerpunkt auf Bevölkerungsgruppen mit einem Risiko für Langzeitpflege und Menschen mit Behinderungen aller Altersgruppen liegen sollte. Der Zugang über Versicherungssysteme und Patientengruppen hinweg sollte beurteilt und verbessert werden, wobei Hindernisse in Bezug auf Anspruchsberechtigung und Kosten beseitigt werden sollten. Dies sollte ein breites Spektrum an Hilfsmitteln wie Mobilitätshilfen, Kommunikationsmittel und digitale Lösungen umfassen, um eine inklusive und umfassende Versorgung zu gewährleisten. Um eine wirksame und nachhaltige Nutzung sicherzustellen, müssen Schulungen und Versorgung angeboten werden.  22) **Aufklären von Gesundheitsdienstleistern sowie Anwenderinnen und Anwendern über die Verfügbarkeit, Verwendung und Vorteile von Hilfsmitteln und Technologien**. Beratung und Unterstützung sollten in allen Bereichen der Gesundheitsversorgung angeboten werden, einschliesslich Krankenhäusern, der Primärversorgung und Langzeitpflegeeinrichtungen. Es ist sicherzustellen, dass Informationen über Hilfsmittel und Technologien für Patientinnen und Patienten, sowie Pflegekräfte leicht zugänglich sind, um eine informierte Nutzung zu unterstützen, die Akzeptanz zu erhöhen und die Selbstverwaltung und Unabhängigkeit zu fördern. |  |

**Considérations politiques et exemples de bonnes pratiques pour renforcer la réadaptation en Suisse**

**VERSION FRANCAISE**

| **Zone d'action WHA** | **Appel de résolution d'origine** | **Considérations politiques** | **Exemples de bonnes pratiques** |
| --- | --- | --- | --- |
| 1. Sensibiliser et susciter un engagement national | Les États membres sont invités à sensibiliser l’opinion publique et à renforcer l’engagement national en matière de réadaptation, notamment en ce qui concerne les technologies d’assistance, et à consolider la planification de la réadaptation, y compris son intégration dans les plans et politiques nationaux de santé, le cas échéant, tout en promouvant le travail interministériel et intersectoriel et la participation significative des usagères et usagers de la réadaptation, en particulier les personnes handicapées, les personnes âgées, les personnes ayant besoin de soins de longue durée, les membres de la communauté et les organisations communautaires et de la société civile à toutes les étapes de la planification et de la mise en œuvre. | 1) **Créer une** **Alliance nationale suisse pour la réadaptation** afin de fédérer les principaux acteurs autour d'un objectif commun : promouvoir le renforcement de la réadaptation en tant que service de santé essentiel parmi les priorités nationales de santé en Suisse. Ces acteurs peuvent inclure les décideurs politiques, les professionnelles et professionnels de la santé, les associations professionnelles de réadaptation, les organismes d'assurance-maladie et sociale, les usagères et usagers des soins, les groupes de défense des droits et les partenaires universitaires. L'Alliance favorisera une compréhension partagée de la définition, du champ d'application et des avantages de la réadaptation, en soulignant son rôle non seulement dans le rétablissement, mais aussi dans la promotion de la participation, l'amélioration des capacités fonctionnelles et le soutien au vieillissement en bonne santé. Un objectif clé de l'Alliance devrait être le lancement d'une campagne nationale de réadaptation inclusive et multilingue destinée au grand public.  2) **Créer une** **Unité de coordination pour la réadaptation** au sein des autorités sanitaires nationales suisses. Cette Unité aura pour objectif principal de renforcer la réadaptation dans le système de santé suisse. Elle servira de source centrale d’information, coordonnera les efforts et soutiendra l’élaboration et la mise en œuvre des stratégies de réadaptation. Elle sera rattachée à l’Office Fédéral de la Santé Publique (OFSP) et/ou à la Conférence suisse des ministres cantonaux de la santé (CDS). Afin d’assurer l’intégration des politiques et du financement de la réadaptation dans les systèmes de santé et de protection sociale, il conviendra d’envisager la participation d’autorités telles que l’Office Fédéral des Assurances Sociales (OFAS), l’Office Fédéral de la Statistique (OFS), le Secrétariat d’État à l’économie (SECO), les Offices cantonaux d’assurance invalidité (IAI) et la Conférence des offices cantonaux d’assurance invalidité (COAI). L’Unité de coordination pour la réadaptation servira également de point de contact avec l’OMS. | - À l'échelle mondiale, il existe une Alliance mondiale pour la réadaptation (WRA) qui est un réseau de parties prenantes engagées à soutenir la mise en œuvre de l'Appel à l'action Réadaptation 2030. (1)  - À l'échelle mondiale également, il existe le programme ReLAB-HS (Learning, Acting and building for Rehabilitation in Health Systems) qui propose des notes d'orientation et des recherches destinées en particulier aux pays à revenu faible et intermédiaire (PRFI). (2)  - En Suisse, il n'existe ni alliance ni unité de coordination pour la réadaptation.  -Pendant la pandémie de COVID-19, la Swiss National COVID-19 Science Task Force a démontré l’utilité d’un conseil scientifique coordonné pour les décisions politiques, soulignant l’intérêt de mécanismes similaires pour renforcer le système de santé suisse. (3) |
| **2. Renforcer le mécanisme de financement** | Les États membres sont invités à mettre en œuvre des moyens appropriés pour renforcer les mécanismes de financement des prestations de réadaptation et de la fourniture d'assistance technique, notamment en intégrant la réadaptation dans les ensembles de soins essentiels lorsque cela est nécessaire. | 3) **Revoir** **les systèmes tarifaires et les régimes de l'assurance-maladie** afin de garantir des prestations de réadaptation équitables, accessibles et durables, adaptés à l’évolution des besoins de la population. Cela implique de lever les obstacles financiers potentiels pour les usagères et usagers des soins de santé, tels que les frais à leur charge, y compris les franchises et les quotes-parts. Les efforts devraient viser une couverture complète en milieu hospitalier, l’étendre aux soins ambulatoires et renforcer le lien entre le financement des services de santé et celui des services sociaux. De plus, des mécanismes de financement adaptés tout au long du parcours de soins pourront ainsi être mis en place.  4) **Promouvoir** **la réadaptation comme un investissement rentable et à forte valeur ajoutée** pour le système de santé. Pour ce faire, il convient d'adopter des modèles de services novateurs tels que la téléréadaptation, la réadaptation à domicile et les approches de soins coordonnés qui mettent l'accent sur la définition d'objectifs et la gestion de cas afin d'améliorer l'efficience. Des études de retour sur investissement (RSI) supplémentaires devraient être menées afin d'évaluer le rapport qualité-prix des différents modèles de réadaptation. Ces évaluations devraient prendre en compte les résultats cliniques et sociétaux, tels que l'amélioration de la participation, la réduction de la charge et le retour au travail, afin de saisir pleinement la valeur générée par les prestations de réadaptation.  5) **Aligne**r **les investissements en réadaptation** sur les projections régionales et cantonales des besoins en soins de santé, en tenant compte des tendances démographiques. Cet alignement devrait s’appuyer sur des indicateurs standardisés et des évaluations des besoins. | - Le nouveau système de financement uniforme des services ambulatoires et hospitaliers (EFAS) offre une bonne opportunité de repérer et de combler les lacunes du financement des soins de santé en Suisse (4).  - Les études sur les résultats sanitaires des modèles innovants comme la téléréadaptation cardiaque hybride menées par l' Inselspital de Berne (5) servent de base pour évaluer le rapport coût-efficacité et le retour sur investissement.  - Afin de permettre une stratégie de financement durable, des évaluations détaillées des besoins doivent être élaborées, comme l'ont montré le canton de Lucerne et la Région de santé commune aux deux Bâles. (6, 7) |
| **3. Étendre à tous les niveaux du système de santé** | Les États membres sont invités à étendre la réadaptation à tous les niveaux de soins de santé, des soins primaires aux soins tertiaires, et à garantir la disponibilité et l’accessibilité financière de prestations de réadaptation de qualité et offerts en temps opportun, accessibles et utilisables par les personnes handicapées, et à élaborer des stratégies de réadaptation communautaires qui permettront à la réadaptation d’atteindre les zones rurales, éloignées et difficiles d’accès mal desservies, tout en mettant en œuvre des stratégies centrées sur la personne et des prestations de réadaptation intensifs, participatifs, spécialisés et différenciés pour répondre aux besoins des personnes ayant des besoins complexes en matière de réadaptation. | 6) **Garantir** **l’accès aux prestations de réadaptation à tous les niveaux de soins** en renforçant les soins primaires, y comprisles médecins généralistes, les infirmiers et les autres professionnelles et professionnels de la santé, et en étendant les services au niveau communautaire (par exemple, la téléréadaptation et les équipes mobiles) et au système de services sociaux. Souligner le rôle de la réadaptation dans la prévention secondaire et tertiaire et la prise en charge des maladies chroniques afin d’améliorer les résultats à long terme et de réduire la pression sur le système de santé. Cet objectif peut être atteint en intégrant la réadaptation aux initiatives cantonales en cours et en améliorant la coordination régionale.  7) **Priorise**r **des prestations de réadaptation abordables, de qualité et accessibles dans toutes les régions**, en s’attachant à réduire les inégalités géographiques et sociales existantes. Accorder une attention particulière aux groupes vulnérables, tels que les personnes âgées (65 ans et plus), les personnes handicapées ou atteintes de maladies chroniques, les personnes sans emploi ou leurs aidants, les personnes vivant en zones rurales ou isolées ou les personnes issues de l’immigration. Garantir un accès équitable aux soins de réadaptation spécialisés, indépendamment du lieu de résidence, du revenu, de la couverture sociale, de la culture, de la langue ou de tout autre obstacle potentiel, notamment en harmonisant les initiatives cantonales et régionales pertinentes. | -L’Office fédéral de la santé publique considère les soins primaires comme un pilier fondamental du système de santé et a élaboré un programme en la matière afin de garantir l’accès à des soins de qualité à tous, dans toutes les régions de Suisse. Ce programme inclut également la prévention, les soins curatifs, la réadaptation et les soins palliatifs. (8) |
| **4. Garantir des interventions intégrées et coordonnées** | Les États membres sont invités à assurer la fourniture intégrée et coordonnée d’interventions de réadaptation de haute qualité, abordables, accessibles, tenant compte des différences entre les sexes, appropriées et fondées sur des données probantes tout au long du continuum de soins, notamment en renforçant les systèmes d’orientation et l’adaptation, la fourniture et l’entretien des technologies d’assistance liées à la réadaptation, y compris après la réadaptation, et en promouvant des environnements inclusifs et sans obstacles. | 8) **Mettre en place** **des mesures et des réglementations visant à harmoniser les prestations de réadaptation en milieu hospitalier, ambulatoire et communautaire tout au long du parcours de soins**. Celles-ci devraient inclure des normes de qualité et des systèmes numériques interopérables afin de faciliter la communication et la coordination entre les différents niveaux de soins. De plus, la gestion des transitions de soins (par exemple, de l'hôpital au domicile, de l'hospitalisation aux soins ambulatoires) et les services d'insertion professionnelle devraient être envisagés afin d'éviter les ruptures de prise en charge.  9) **Investir dans** **des mécanismes de soutien solides pour garantir des soins de réadaptation de haute qualité**, en s'appuyant sur le travail de la Commission fédérale de la qualité (CFQ) et d'initiatives connexes, telles que l'Association nationale pour le développement de la qualité dans les hôpitaux et cliniques (ANQ). Ces mécanismes peuvent inclure des programmes de formation, des systèmes d'audit et des outils de retour d'information des patients. Les indicateurs de qualité doivent être spécifiques à la réadaptation et refléter des résultats centrés sur le patient, notamment l'opérationnalisation et la mesure des objectifs de participation. | - Des mesures de qualité bien établies comme l'ANQ (9) ou les critères de qualité pour la réadaptation de Swiss Reha (10) sont nécessaires pour assurer un niveau élevé de prestations de réadaptation et de soins de santé.  - Des projets comme ParaWork (11) ou ParaWG (12) du Centre suisse des paraplégiques montrent l'importance de prendre en compte la continuité des soins ainsi que la réintégration professionnelle et sociale. |
| **5. Développer des compétences en réadaptation multi­disciplinaires** | Les États membres sont invités à développer de solides compétences multidisciplinaires en matière de réadaptation adaptées au contexte national, y compris pour l'ensemble des professionnelles et professionnels de la santé concernés ; à renforcer leurs capacités d'analyse et de prévision des pénuries de main-d'œuvre et à promouvoir la formation initiale et continue des professionnels et du personnel travaillant dans les prestations de réadaptation ; et à reconnaître et à répondre aux différents types de besoins en matière de réadaptation, tels que les besoins liés au fonctionnement physique, mental, social et professionnel, notamment par l'intégration de la réadaptation dans la formation initiale des professionnelles et professionnels de la santé, afin que les besoins en réadaptation puissent être identifiés à tous les niveaux de soins. | 10) **Intégrer** **la réadaptation au tronc commun de toutes les professions de santé, de la formation initiale à la formation continue et spécialisée**. Cela devrait inclure les connaissances fondamentales pour tous les professionnelles et professionnels de la santé, ainsi que des compétences avancées pour ceux qui travaillent directement en réadaptation. Mettre l'accent sur la formation en réadaptation multidisciplinaire et interprofessionnelle, en particulier pour les médecins généralistes, qui constituent souvent un point d'entrée clé dans le système de réadaptation. Améliorer l'efficacité des équipes de réadaptation multidisciplinaires en renforçant les compétences en gestion de cas et en coordination afin d'assurer la continuité des soins et une collaboration efficace entre les différents lieux de soins. Ces composantes pédagogiques devraient être intégrées de manière structurelle grâce à une collaboration avec les établissements d'enseignement supérieur et les associations professionnelles.  11) **Remédier** **à la pénurie de main-d’œuvre en assurant un nombre suffisant de professionnels qualifiés dans toutes les disciplines de la réadaptation**, en accordant une attention particulière aux besoins d’une population vieillissante et à l’augmentation des maladies chroniques.  Appuyer le développement d’outils nationaux de suivi et de prévision des effectifs pour une planification fondée sur des données probantes et envisager la mise en œuvre de programmes incitatifs (bourses d’études, stages et possibilités de perfectionnement professionnel, par exemple) afin d’attirer et de fidéliser les professionnels de la réadaptation, notamment dans les zones mal desservies. De plus, soutenir l’agrément et la formation des prestataires formés à l’étranger afin de renforcer les effectifs en réadaptation.  12) **Améliorer** **la fidélisation et les conditions de travail afin de maintenir un personnel de réadaptation stable, en bonne santé et qualifié** . Cela comprend le développement de nouveaux modèles de travail attrayants et flexibles, incluant des options pour les professionnels qui reprennent leur activité. Le suivi de la satisfaction du personnel et des taux de rotation peut contribuer à l'amélioration continue et soutenir le développement durable de la main-d'œuvre. | - Face aux changements envisagés dans le domaine des soins de santé, notamment une prise en charge plus intégrée, un traitement individualisé des patients polymorbides et le vieillissement de la population, le canton de Berne et la Haute École spécialisée bernoise prévoient que le système de santé sera plus interdisciplinaire, ce qui nécessitera une formation adaptée . (13)  - Le programme de collaboration en matière d'éducation interprofessionnelle constitue un bon exemple de développement de programmes d'études visant à promouvoir l'apprentissage interprofessionnel en vue de la préparation au travail d'équipe dans les soins aux patients. (14)  En Suisse, il existe des certificats d’études supérieures axés sur le travail interprofessionnel dans le domaine de la santé, comme par exemple le CAS en gestion de la réadaptation à l’Université de Lucerne. Les hautes écoles spécialisées telles que l’Université des sciences appliquées de Zurich (ZHAW) intègrent la formation interprofessionnelle dans les cursus de licence des professions de santé comme les soins infirmiers, la physiothérapie, l’ergothérapie, la sage-femme et la prévention et la promotion de la santé. (15) |
| **6. Améliorer les systèmes d'information et la collecte de données** | Les États membres sont invités à renforcer leurs systèmes d’information sanitaire afin de recueillir des informations pertinentes pour la réadaptation, notamment des données de réadaptation au niveau du système et des informations sur le fonctionnement, en utilisant la Classification internationale du fonctionnement, du handicap et de la santé, en veillant à la désagrégation des données par sexe, âge, handicap et tout autre facteur contextuel pertinent, et au respect de la législation sur la protection des données, pour un suivi rigoureux des résultats et de la couverture de la réadaptation. | 13) **Investir dans les systèmes d'information sanitaire afin d'améliorer la collecte, le partage et l'utilisation systématique des données relatives à la réadaptation, y compris les informations fonctionnelles.** Le fonctionnement, tel que défini par la Classification Internationale du Fonctionnement, du handicap et de la santé (CIF) décrit l'expérience vécue de la santé par une personne. Elle fait référence aux capacités de santé intrinsèques d'un individu et à ses performances réelles dans des contextes concrets, opérationnalisées à travers les domaines des fonctions et structures corporelles, des activités et de la participation, en interaction avec des facteurs contextuels. Explorer le potentiel de l'intelligence artificielle pour faciliter la collecte et l'analyse des données, la prise de décision et l'amélioration des services. Cela implique de garantir une utilisation critique, responsable et éthique de l'IA, en accordant une importance particulière à la protection et la confidentialité des données.  14) **Promouvoir la reconnaissance du fonctionnement comme troisième indicateur de santé fondamental, au même titre que la morbidité et la mortalité,** en fournissant des données probantes et en favorisant le consensus entre les parties prenantes**.** Indicateur précieux de la santé de la population et composante essentielle d'un suivi rigoureux des résultats de la réadaptation, le fonctionnement devrait être intégré aux systèmes nationaux d'information sanitaire et aux statistiques nationales.  15) **Combler** **les lacunes en matière d’infrastructure numérique en garantissant la sécurité des données et en encourageant l’adoption du système national de dossier électronique du patient (DEP)** qui rend compte d’un ensemble essentiel d’indicateurs et de résultats de réadaptation de manière unifiée et interopérable. Il convient de soutenir les solutions abordables, telles que les plateformes libres ou les subventions pour les petits établissements, afin de faciliter une large adoption. | L’hôpital pour enfants de Lucerne collabore avec le Centre d’analyse de la santé infantile (CCHA) pour le traitement et la santé des enfants en analysant les données de santé collectées de façon systématique. Les données concernant les patientes et patients adultes sont collectées à l’aide d’outils tels que ANQ (9), le dossier médical électronique eHealth Suisse (16), et données gouvernementales sur l'emploi (17), et sert de réseau numérique pour les soins de santé (16). |
| **7. Promouvoir la recherche en réadaptation** | Les États membres sont invités à promouvoir la recherche de haute qualité en matière de réadaptation, notamment la recherche sur les politiques et les systèmes de santé. | 16) **Elargir** **la recherche de haute qualité en réadaptation aux niveaux des systèmes, des services et pratiques cliniques afin** d’évaluer l’efficacité et le rapport coût-efficacité des interventions. Cela comprend la recherche sur la mise en œuvre pour combler l’écart entre les données probantes et la pratique, ainsi que l’élaboration et l’adoption d’outils standardisés et centrés sur le patient afin d’améliorer la coordination des cas tout au long du continuum de soins et de permettre la documentation systématique des résultats de la réadaptation, y compris les objectifs individuels.  17) **Faire** **des sciences de la réadaptation un axe académique prioritaire** avec des chaires de professeur titulaire dédiées aux sciences de la réadaptation et à différentes disciplines professionnelles, telles que la médecine de réadaptation, la physiothérapie, l’ergothérapie, etc.  18) **Renforcer la base de données factuelles en matière de réadaptation et mettre l’accent sur la transposition des résultats de la recherche en politiques de santé,** par le biais de lignes directrices nationales, de groupes consultatifs d’experts et de stratégies structurées de transfert des connaissances. Il s’agit également de soutenir la recherche sur les systèmes et les politiques de santé afin d’éclairer la prise de décision, notamment grâce à des mécanismes de financement de la recherche dédiés.  19) **Promouvoir la collaboration nationale et mettre en place des réseaux de recherche en réadaptation**, incluant cantons, universités, assureurs et autres acteurs, afin d’accroître leur portée et leur impact. Intégrer les initiatives existantes en sciences et gestion de la réadaptation des universités et des hautes écoles spécialisées dans leurs programmes multidisciplinaires. | - À l’échelle mondiale, l’Alliance de l’OMS pour la recherche en politiques et systèmes de santé encourage la production et l’utilisation de données probantes pour renforcer les systèmes de santé, offrant un modèle pour le développement de la recherche et du transfert de connaissances en réadaptation. (18) |
| **8. Intégrer dans la préparation et la réponse aux situations d'urgence** | Les États membres sont invités à veiller à l’intégration rapide de la réadaptation dans les dispositifs de préparation et de réponse aux situations d’urgence, notamment au sein des équipes médicales d’urgence. | 20) **Intégrer pleinement** **la réadaptation aux plans d’intervention d’urgence et aux stratégies de préparation**, en privilégiant les outils numériques pour une coordination en temps réel pendant et après les crises. Afin d’assurer la continuité des soins pendant et après les urgences de santé publique, la réadaptation doit être un élément essentiel des comités de gestion des catastrophes. Les professionnels de la réadaptation doivent participer aux formations, aux simulations et aux exercices d’urgence. De plus, la réadaptation doit être intégrée aux processus d’adaptation à long terme mis en œuvre après les crises, les urgences de santé publique ou autres situations d’urgence. |  |
| **9. Investir dans les technologies d'assistance** | Les États membres sont invités à inciter les acteurs publics et privés à stimuler l'investissement dans le développement de technologies d'assistance disponibles, abordables et utilisables, ainsi que dans le soutien à la recherche et à l'innovation en matière de mise en œuvre, pour une prestation efficace et un accès équitable, en vue de maximiser l'impact et le rapport coût-efficacité. | 21) **Intensifier** **les efforts visant à garantir un accès équitable à des aides techniques et technologiques abordables, ainsi qu'à des aménagements du milieu de vie,** afin d'améliorer le fonctionnement au quotidien et l'autonomie. L'accès devrait reposer sur les besoins individuels plutôt que sur un diagnostic, en accordant une attention particulière aux populations à risque de nécessiter des soins de longue durée et aux personnes handicapées de tous âges. Évaluer et renforcer l'accès à ces aides dans le cadre des régimes d'assurance et pour les différents groupes de patients, en supprimant les obstacles liés à l'admissibilité et au coût. Ceci devrait inclure un large éventail d'aides techniques, telles que les aides à la mobilité, les outils de communication et les solutions numériques, afin de garantir un soutien inclusif et complet. Une formation et un accompagnement des utilisateurs doivent être proposés pour assurer une utilisation efficace et durable.  22) **Sensibiliser les professionnelles de la santé et les usagères et usagers aux dispositifs et technologies d’assistance disponibles, à leur utilisation et à leurs avantages.** Un accompagnement et un soutien doivent être proposés dans tous les contextes de soins, y compris les hôpitaux, les soins primaires et les établissements de soins de longue durée. Veiller à ce que l’information sur les dispositifs et technologies d’assistance soit facilement accessible aux patients et à leurs aidants afin de favoriser une utilisation éclairée, une meilleure acceptation et promouvoir l’autonomie et l’indépendance. |  |

**References Best Practice Examples**

1. World Health Organization. World Rehabilitation Alliance: What is the World Rehabilitation Alliance? Geneva: WHO; 2025 [cited 2026 August 28]. Available from: <https://www.who.int/initiatives/world-rehabilitation-alliance>
2. RelabHS. Who we are. Geneva: World Health Organization; 2025 [cited 2026 August 28]. Available from: <https://relabhs.org/who-we-are/>
3. Swiss National COVID-19 Science Task Force (ncs-tf). Abschlussbericht der Swiss National COVID-19 Science Task Force (ncs-tf). Bern: ncs-tf; 2022 Mar 29 [cited 2026 August 28]. Available from: <https://sciencetaskforce.ch/wp-content/uploads/2022/04/AbschlussberichtSTF29Mar2022-DE.pdf>
4. Strehle O, Müller A. EFAS und die Integrierte Versorgung – zukunft beginnt. Bern: Forum Für Integrierte Versorgung. Competence; 2024 [cited 2026 August 28]. Available from: [https://fmc. ch/wp-content/uploads/2024/04/Artikel-und-Studien_Artikel-Competence-EFAS- und-IntV.pdf.](https://fmc.ch/wp-content/uploads/2024/04/Artikel-und-Studien_Artikel-Competence-EFAS-und-IntV.pdf)
5. Eser P, Pini M, Vetsch T, Marcin T, Berni S, Burri R, et al. Comparison of patient characteristics and health outcomes between self-selected centre-based cardiac rehabilitation and hybrid cardiac telerehabilitation: a prospective cohort study. Eur J Prev Cardiol. 2025;32(3):303–14. doi:10.1093/eurjpc/zwae061.
6. Kanton Luzern. Planungsbericht über die Gesundheitsversorgung im Kanton Luzern 2024. Luzern: Gesundheits- und Sozialdepartement; 2024 Aug 19. Report No. B36 [cited 2026 August 28]. Available from: <https://gesundheit.lu.ch/-/media/Gesundheit/Dokumente/Gesundheitsversorgung/Planungsbericht/Planungsbericht_ber_die_Gesundheitsversorgung_im_Kanton_Luzern_2024.pdf?rev=3b8ae302f1a44cb2adc4e55fd46cfefa>
7. Gesundheitsdepartement Basel-Stadt & Gesundheitsdirektion Basel-Landschaft. Versorgungsplanungsbericht 2023: Gemeinsame Gesundheitsregion – Rehabilitation. Basel: Gesundheitsdepartement Basel-Stadt und Basel-Landschaft; 2023 Sep 6 [cited 2026 August 28]. Available from: <https://media.bs.ch/original_file/bf31b5e8ecfa0e90ebbae1a50e68fa4001e643dd/versorgungsplanungsbericht-rehabilitation-ggr-2023-0.pdf>
8. Bundesamt für Gesundheit (BAG). Agenda Grundversorgung. Bern: BAG; 2025 [cited 2026 August 28]. Available from: <https://www.bag.admin.ch/de/agenda-grundversorgung>
9. ANQ – Nationaler Verein für Qualitätsentwicklung in Spitälern und Kliniken. ANQ – das Kompetenzzentrum für Qualitätsmessungen in Spitälern und Kliniken. Bern: ANQ; 2025 [cited 2026 August 28]. Available from: <https://www.anq.ch/de/>
10. Swiss Reha. Qualitäts- und Leistungskriterien für die ambulante und teilstationäre Rehabilitation. Aarau: Swiss Reha; 2016 [cited 2026 August 28]. Available from: <https://www.swiss-reha.com/api/rm/U573455FY3C2X3D/swiss-reha-d-qualitaets-leistungskr-a5-dt.pdf>
11. Schweizer Paraplegiker-Zentrum (SPZ). ParaWork. Nottwil: SPZ; 2025 [cited 2026 August 28]. Available from: <https://www.paraplegie.ch/spz/de/ueber-uns/partizipation/parawork/>
12. Schweizer Paraplegiker-Zentrum (SPZ). ParaWG Wohntraining – die Erfolgsstory. Nottwil: SPZ; 2025 [cited 2026 August 28]. Available from: <https://www.paraplegie.ch/spz/de/ueber-uns/soziale-und-berufliche-integration/parawg-wohntraining-die-erfolgsstory/>
13. Berner Fachhochschule (BFH). Forschungsprojekt «Panorama Gesundheitsberufe 2030»: Kompetenzen – Personal – Aus- und Weiterbildung. Bern: BFH; 2013 [cited 2026 August 28]. Available from: <https://www.bfh.ch/dam/jcr:01c59dc4-927d-4289-b317-64e6891f2504/Flyer_D_Forschungsprojekt_Panorama_Gesundheitsberufe_2030.pdf>
14. Interprofessional Education Collaborative (IPEC). About us. Washington, DC: IPEC; 2025 [cited 2026 August 28]. Available from: <https://www.ipecollaborative.org/about-us>
15. Zürcher Hochschule für Angewandte Wissenschaften (ZHAW). Bachelorstudiengänge Gesundheit. Winterthur: ZHAW; 2022 [cited 2025 Jul 31]. Available from: <https://www.zhaw.ch/storage/gesundheit/ueber-uns/info-broschueren/BSc/broschuere-bsc-studiengaenge-zhaw-gesundheit.pdf>
16. E-Health Suisse. Digital Health Data – standardised, secured, shared. Bern: E-Health Suisse; 2025 [cited 2026 August 28]. Available from: <https://www.e-health-suisse.ch/>
17. Bundesamt für Sozialversicherungen (BSV). Bundesamt für Sozialversicherungen BSV. Bern: BSV; 2025 [cited 2026 August 28]. Available from: <https://www.bsv.admin.ch/bsv/de/home.html>
18. Alliance for Health Policy and Systems Research. Aiming for impact: 2024–2028 strategy of the Alliance for Health Policy and Systems Research. Geneva: World Health Organization; 2024 [cited 2026 August 28]. Available from: <https://ahpsr.who.int/publications/i/item/aiming-for-impact>
